# Tumor response from durvalumab (MEDI4736) + tremelimumab treatment in patients with advanced non-small cell lung cancer (NSCLC) is observed regardless of PD-L1 status

**DOI:** 10.1186/2051-1426-3-S2-P193

**Published:** 2015-11-04

**Authors:** Naiyer Rizvi, Jamie Chaft, Ani Balmanoukian, Sarah B Goldberg, Rachel E Sanborn, Keith E Steele, Marlon C Rebelatto, Yu Gu, Joyson J Karakunnel, Scott Antonia

**Affiliations:** 1 Columbia University Medical Center, New York, NY, USA; 2 Memorial Sloan Kettering Cancer Center, New York, NY, USA; 3 The Angeles Clinic and Research Institute, Los Angeles, CA, USA; 4 Yale University, Yale Cancer Center, New Haven, CT, USA; 5 Earle A. Chiles Research Institute, Providence Cancer Center, Portland, OR, USA; 6 MedImmune, Gaithersburg, MD, USA; 7 Moffitt Cancer Center, Tampa, FL, USA

## Background

As single agents, durvalumab (MEDI4736), a human IgG1 anti-PD-L1 antibody, and tremelimumab, a human IgG2 anti-CTLA-4 antibody, have shown acceptable safety profiles and antitumor activity. Similar to other anti-PD-L1/anti-PD-1 monotherapies, durvalumab has shown greater objective tumor response rates in PD-L1-positive patients compared with PD-L1-negative patients. Anti-CTLA4 therapies activate T-cells and may increase immune infiltrate and PD-L1 expression in tumor cells and tumor infiltrating immune cells. Thus, combination therapy with durvalumab and tremelimumab could be active in NSCLC regardless of baseline PD-L1 expression.

## Methods

This is a phase 1, open-label, dose-escalation/expansion study (NCT02000947) of D+T in patients with Stage III/IV NSCLC (any number of prior lines of therapy; immunotherapy-naïve). The primary endpoint is safety and tolerability; secondary endpoints include investigator-reported RECIST v1.1 response. PD-L1 expression was tested retrospectively using an immunohistochemical assay (Ventana).

## Results

As of 1 June 2015, 102 patients received treatment in the dose escalation phase; combinations of durvalumab [3 mg/kg (D3) to 20 mg/kg (D20) every 2 (q2w) or 4 weeks (q4w)] and tremelimumab [1 mg/kg (T1) to 3 mg/kg (T3)] q4w, plus a D15 + T10 combination, were explored. Across all cohorts, 80% and 42% of patients had ≥1 treatment-related AE (any Grade and Grade 3/4, respectively); 28% discontinued treatment due to a related AE. A greater frequency of AEs, without a corresponding increase in tumor response, was seen with increasing T dose. In the combined T1 cohort (D10–D20), 73% and 30% of patients had ≥1 related AE (any Grade and Grade 3/4, respectively); 16% discontinued treatment due to a related AE. There were 3 treatment-related deaths (myasthenia gravis, T1; pericardial effusion, T1; neuromuscular disorder, T3).

84 patients (73 EGFR/ALK wild-type; 77 non-squamous; 48 with ≥2 prior lines of therapy) were evaluable for response (Table 1). The overall response rate (confirmed+unconfirmed) was 25%. Higher response rates were observed in those with 1 vs ≥2 prior therapies. Response rates do not appear dependent on PD-L1 status: 35% (PD-L1-positive), 22% (PD-L1-negative, <25% tumor cell staining) and 33% (PD-L1-negative, 0% tumor cell staining). Similar findings were observed for the combined T1 cohort. D+T also showed good durability of response similar to that seen for monotherapy.

**Table 1:** Response rates (Confirmed/unconfirmed with ≥16 weeks follow-up)

	Overall population				EGFR/ALK wild-type population			
	All cohorts		Combined cohort: D10–20 q4w or q2w + T1		All cohorts		Combined cohort: D10–20 q4w or q2w + T1	

	n/N (%)	95% CI	n/N (%)	95% CI	n/N (%)	95% CI	n/N (%)	95% CI

All patients	21/84 (25)	16–36	11/39 (28)	15–45	21/73 (29)	19–41	11/34 (32)	17–51
PD-L1^+^ (≥25%)	7/20 (35)	15–59	3/9 (33)	8–70	7/17 (41)	18–67	3/9 (33)	8–70
PD-L1^-^ (<25%)	11/49 (22)	12–37	6/23 (26)	10–48	11/45 (24)	13–40	6/19 (32)	13–57
PD-L1^-^ (0%)	9/27 (33)	17–54	6/12 (50)	21–79	9/26 (35)	17–56	6/11 (55)	23–83
All 2L patients	15/32 (47)	29–65	7/16 (44)	20–70	15/31 (48)	30–67	7/15 (47)	21–73
PD-L1^+^ (≥25%)	6/8 (75)	35–97	2/3 (67)	9–99	6/8 (75)	35–97	2/3 (67)	9–99
PD-L1^-^ (<25%)	7/18 (39)	17–64	4/11 (36)	11–69	7/17 (41)	18–67	4/10 (40)	12–74
PD-L1^-^ (0%)	6/8 (75)	35–97	4/5 (80)	28–100	6/8 (75)	35–97	4/5 (80)	28–100

2L, second line: 1 prior line of therapy, receiving D+T in second line

## Conclusions

D+T at selected phase 3 dose (D20, T1) has a manageable tolerability profile and anti-tumor activity in NSCLC. Unlike anti- PD-1/PD-L1 monotherapies, the combination of D+T appears to be active regardless of PD-L1 status, including even in patients with no tumor cell membrane PD-L1 staining, a setting where patients would not be expected to derive significant benefit from anti-PD-1/PD-L1 monotherapy over current standard of care [1,2].
